# Construction and validation of indicators for nursing management in blood transfusion

**DOI:** 10.1590/0034-7167-2024-0229

**Published:** 2025-07-11

**Authors:** Daiana de Mattia, Dulcinéia Ghizoni Schneider, Francine Lima Gelbcke, Maria Manuela Frederico Ferreira, Edson Pacheco Paladini, Jose Luís Guedes dos Santos, Marcela Ganzella Sisdelli

**Affiliations:** I Universidade Federal de Santa Catarina. Florianópolis, Santa Catarina, Brazil; II Escola Superior de Enfermagem de Coimbra. Coimbra, Portugal; III Fundação Hemocentro de Ribeirão Preto. Ribeirão Preto, São Paulo, Brazil

**Keywords:** Blood Transfusion, Nursing Care, Management Indicators, Validation Study, Hemotherapy Service, Transfusión Sanguínea, Atención de Enfermería, Indicadores de Gestión, Estudio de Validación, Servicio de Hemoterapia

## Abstract

**Objectives:**

to construct and validate indicators for nursing management in blood transfusion.

**Methods:**

methodological study that involved the creation of technical sheets of indicators for nursing management in blood transfusion and their validation by 17 nurses working in Brazilian transfusion agencies. Data analysis was performed using descriptive statistics, content validity index and calculation of the percentage of agreement.

**Results:**

27 indicators were validated for nursing management in blood transfusion, with a content validity index higher than 0.80 and a percentage of agreement higher than 80%. These indicators were organized into the categories structure, process and results, according to the environment evaluated.

**Conclusions:**

the established indicators present evidence of content validity, allowing their application in nursing management in the transfusion process, promoting continuous improvement of nursing team practices and strengthening transfusion safety.

## RESUMEN

**Objetivos:**

construir y validar indicadores para la gestión de enfermería en transfusión sanguínea.

**Métodos:**

estudio metodológico que involucró la creación de fichas técnicas de indicadores para la gestión de enfermería en transfusión sanguínea y su validación por 17 enfermeros que actúan en agencias de transfusión brasileñas. El análisis de datos se realizó mediante estadística descriptiva, índice de validez de contenido y cálculo del porcentaje de acuerdo.

**Resultados:**

se validaron 27 indicadores para la gestión de enfermería en transfusión sanguínea, con un índice de validez de contenido mayor a 0,80 y un porcentaje de concordancia mayor al 80%. Estos indicadores se organizaron en las categorías estructura, proceso y resultados, según el entorno evaluado.

**Conclusiones:**

los indicadores establecidos presentan evidencia de validez de contenido, permitiendo su aplicación en la gestión de enfermería en el proceso transfusional, promoviendo la mejora continua de las prácticas del equipo de enfermería y fortaleciendo la seguridad transfusional.

## INTRODUCTION

Quality is a dynamic, public domain concept that encompasses multiple elements with varying levels of importance, the primary objective of which is to meet consumer needs^(1)^. In the healthcare context, it is associated with user satisfaction, considering the delivery of effective and safe care with a high level of professional excellence, while respecting prevailing cultural and social values^(2)^.

To meet these requirements, continuous assessments of healthcare processes are essential to identify factors that directly impact the work of professionals responsible for patient care^(3)^. As important as producing quality is evaluating it, as thorough analysis enables the monitoring of ongoing actions to determine whether the objectives and expected outcomes are being achieved^(1)^.

Quality assessment is one of the main tools for meeting the informational needs of managers, proving essential for reviewing and redirecting strategies implemented in healthcare actions^(4)^. One of the pioneers in healthcare quality publications developed a conceptual model for evaluation that has been widely adopted in health services. This model is based on three domains: structure, process, and outcomes^(5)^.

Structure pertains to the more stable aspects of healthcare delivery, including the physical, human, material, and financial resources required for care provision. Process involves the actions undertaken during care, encompassing both healthcare professionals and patients, always based on predefined standards. Outcomes refer to the effects of care, considering patient health, adherence to standards, and fulfillment of expectations^(5)^.

Indicators serve as an evaluation tool; they are measurable representations, or quantitative data, that reflect the characteristics of products and processes, making them essential for monitoring and improving organizational performance and quality^(1)^. Indicators form the foundation for critical analysis of results, aiding in decision-making, planning, and process control, and guiding necessary improvements^(6)^.

Healthcare management processes are widely applied in nursing practice. However, for nurses to develop effective tools to evaluate care outcomes, they must rely on data that directly or indirectly reflect the reality of this care^(3)^. Therefore, it is crucial that these professionals have access to indicators sensitive to their work, which reflect the structure, process, and outcomes of the care provided^(7)^. Furthermore, it is essential to strengthen the culture of quality within services by promoting training for the creation and analysis of indicators. This approach facilitates a continuous and dynamic evaluation of care, focusing on patient safety, reducing adverse events, and improving management processes^(8)^.

Authors^(9)^ state that nurses in hemotherapy must play a crucial role in meeting the needs of blood recipients, whether by constantly striving to provide quality services and products or by delivering quality care. This role also extends to the production of blood components, the development of teaching and research, and narrowing the gap between practice and scientific knowledge.

Legally, hemotherapy services in Brazil are required to implement indicators and goals to monitor the performance of processes throughout the entire blood cycle, from collection to transfusion^(10)^. However, a study revealed a scarcity of indicators related to care provided to patients undergoing blood transfusion, both nationally and internationally, highlighting the need for further research on this topic^(11)^.

## OBJECTIVES

To develop and validate indicators for nursing management in blood transfusion.

## METHODS

### Ethical aspects

The study was conducted in accordance with the ethical guidelines established by national and international regulatory bodies and was approved by the Research Ethics Committee of the Federal University of Santa Catarina and the Oncology Research Center (CEPON in Portuguese). All participants signed the Informed Consent Form (ICF).

### Study Design, Period, and Location

This methodological study aimed to develop and validate indicators for nursing management in the transfusion process. The study followed the methodological procedures proposed by McGlynn and Asch^(12)^, encompassing four steps: selecting the areas of evaluation, choosing the indicators, designing the specifications for a measure, and testing the scientific strength of the measure.

Data collection was conducted online between February and April 2023 in transfusion agencies located across the five regions of Brazil.

### Population, inclusion, and exclusion criteria

A total of 174 nurses working in transfusion agencies across Brazil’s five regions were intentionally invited to participate as expert judges. The inclusion criteria required nurses to have at least one year of active employment in a transfusion agency. Nurses who were on leave or vacation during the data collection period were excluded from the study.

### Study protocol

The first two steps of the study-selecting the areas of evaluation and choosing performance indicators-were detailed in two manuscripts, one of which has been published in a national journal^(11)^. From these steps, 27 indicators for nursing management in blood transfusion were defined.

In the third step, specifications for a measure were developed by constructing technical data sheets for the indicators. These were adapted from the model recommended by Paladini^(1)^ and included the following elements: Objective: Defines what the indicator is intended to evaluate; Justification: Explains the importance of conducting the evaluation proposed by the indicator; Environment: Describes the nature of the indicator; Standard: Specifies the reference used to assess whether the process showed improvement; Description: Details the context, situations, or nature that essentially characterizes the indicator; Factor: Explains how the indicator is calculated; Measure: Defines the unit used to measure factors, including the international measurement system and frequency of verification.

In the fourth step, the scientific strength of the measure was tested through content validity. Data collection was conducted using an online form created on the Google Forms^®^ platform. The form link was sent via email along with an invitation letter. The form was divided into the following sections: 1) ICF: For reading and confirming consent to participate in the study; 2) Expert Judges’ Profile: Comprised 14 questions about the professional and their workplace; 3) Evaluation of the 27 Indicators: Included their respective technical data sheets.

In Section 3, closed-ended questions were used to evaluate the spelling, relevance, objectivity, clarity, precision, feasibility, representativeness, visualization, adjustment, uniqueness, scope, and results of the indicators, as recommended by Paladini^(1)^. Additionally, the importance of each indicator for nursing management was assessed.

The questionnaire used a four-point Likert scale (Strongly Disagree to Strongly Agree). Space was also provided for participants to leave comments and/or suggestions to refine the indicators.

In the first round, 17 judges completed the questionnaire within the 30-day deadline. For the second round, the content of nine indicators was reformulated, and a new indicator was created based on the judges’ recommendations. The revised form, incorporating these changes, was sent to the same group of judges, with a 20-day deadline for responses. Seven judges participated in this round, providing further suggestions and observations to improve the indicators.

### Data analysis and statistics

The data submitted by the judges were stored in a Microsoft Excel^®^ spreadsheet for subsequent analysis, which was performed using the Statistical Package for the Social Sciences (SPSS) version 23 (IBM Corporation, Armonk, NY, USA).

The normality of the data was assessed using the Shapiro-Wilk test. For numerical variables, descriptive statistics were applied, including measures of central tendency and dispersion, while categorical variables were presented as absolute and relative frequencies.

To evaluate consensus among the expert judges across the two rounds, two methods were employed: the Content Validity Index (CVI) and the Percentage Agreement. The CVI was calculated as the ratio of the sum of “3” and “4” responses to the total number of participating judges. The Percentage Agreement was calculated as the ratio of the sum of concordant responses to the total number of participating judges, multiplied by 100. The literature recommends that, for an item or the overall questionnaire to be considered acceptable, a CVI of 0.8 and 80% agreement among participants must be achieved^(13,14)^.

## RESULTS

The data from the first round were obtained from a panel of expert judges consisting of 17 nurses. The minimum recorded age was 28 years, and the maximum was 55 years, with a mean age of 41.0 (SD = 7.9) years. Fourteen (82.4%) held a degree in Nursing; two (11.8%) had degrees in Nursing and Obstetrics; and one (5.9%) held degrees in Nursing and Nephrology. Eleven participants held a specialization title, and six (35.3%) had a master’s degree. The average length of academic training was 14.6 (SD = 10.0) years, and the median length of time working in a transfusion agency was 4.0 (2.0 - 8.0) years.

Regarding the characteristics of the Transfusion Agencies where the judges work, key data include: eight (47.1%) were from the South region; 14 (82.4%) were public; ten (58.8%) were managed by a hospital/clinic; and 11 (64.7%) agencies performed 100 to 500 blood transfusions per month. Seven (41.2%) agencies held some form of accreditation, including: Joint Commission International, International Organization for Standardization (ISO) 9001, and National Accreditation. Two agencies had multiple accreditations; two held both ISO and American Association of Blood Banks (AABB) accreditations, and 15 (88.2%) used some form of indicator to evaluate the work process.

All indicators had a total CVI above 0.80, with values ranging from 0.85 to 1.00 for the evaluated items. Regarding the percentage of agreement, all indicators achieved more than 80% agreement among the expert judges, ranging from 85% to 100%. In terms of importance, of the 27 indicators, 17 were rated as very important or important, 10 were considered of little importance by one or two judges, and only two were rated as unimportant by one judge.

The judges provided several comments and suggestions, which were analyzed. It was concluded that nine indicators required refinement.

Considering this reformulation, although the indicators achieved a CVI > 0.80 and a percentage of agreement > 80%, a second round with the expert judges was conducted to reassess the CVI of the revised indicators and the newly created indicator. Seven nurses participated in the second round and reassessed the following: Agreement and Spelling; Relevance; Objectivity; Clarity; Precision; Feasibility; Representativeness; Visualization; Adjustment; Uniqueness; Scope; and Results of the ten reformulated indicators.

The total CVI for the indicators was greater than 0.80, ranging from 0.93 to 1.00, and the percentage of agreement ranged from 93% to 100%, demonstrating that all indicators exceeded the minimum acceptable threshold according to the literature^(13,14)^.

Some participants made comments and suggestions regarding specific indicators. However, these were not accepted as they contradicted current legislation, undermined the objective of the proposed indicator, or were redundant with an existing indicator.

As for the new indicator, it was evaluated for its relevance to nursing management in the transfusion process and was considered relevant by the seven judges who assessed it.

At the end of the second round, 27 indicators for nursing management in blood transfusion were validated. These indicators are organized according to the environment^(5)^ and are presented in Chart 1.

**Chart 1:** Validated Indicators for Nursing Management in Blood Transfusion, Florianópolis, Santa Catarina, Brazil, 2024

ENVIRONMENT	INDICATORS
**Structure**	Indicator 1 - Number of blood component transfusion requests received by the transfusion agency
	Indicator 8 - Blood component discard rate during visual inspection
	Indicator 21 - Blood component discard rate at the transfusion agency
	Indicator 22 - Number of nonconformities identified in the evaluation of materials and reagents quality
	Indicator 23 - Rate of critical equipment with qualification, calibration, and preventive maintenance performed
	Indicator 24 - Rate of professionals who received training on the blood transfusion process at the institution
	Indicator 25 - Rate of blood components with no record of final destination
	Indicator 26 - Number of meetings held by the transfusion committee
**Process**	Indicator 2 - Rate of transfusion requests received by the transfusion agency with incomplete documentation
	Indicator 3 - Rate of transfusion requests received that do not comply with the clinical indication for each blood component
	Indicator 4 - Number of discrepancies between blood typing results found in pre-transfusion tests and the results recorded in the patient’s history
	Indicator 5 - Rate of incidents related to blood samples for pre-transfusion tests
	Indicator 6 - Rate of incidents related to pre-transfusion tests
	Indicator 9 - Rate of discrepant blood typing results found in the retyping of red blood cell concentrates released for transfusion
	Indicator 10 - Index of compatible red blood cell concentrates and those effectively transfused
	Indicator 11 - Number of blood components with inconsistencies in the release label
	Indicator 13 - Rate of blood transfusions without dual-checking recorded before the initiation of the blood component installation
	Indicator 14 - Rate of transfusions performed without verification of vital signs before starting the transfusion
	Indicator 27 - Rate of active search for transfusion reactions
**Result**	Indicator 7 - Rate of compliance with compatibility recommendations during blood component selection
	Indicator 12 - Rate of incidents related to patient preparation for blood transfusion
	Indicator 15 - Rate of incidents related to improper patient identification
	Indicator 16 - Rate of patients not monitored during blood transfusion
	Indicator 17 - Index of transfusion reactions by type, severity, blood component type, and time of onset
	Indicator 18 - Rate of nonconformities related to blood transfusion records in the patient’s chart
	Indicator 19 - Rate of nonconformities related to blood transfusion records at the transfusion agency
	Indicator 20 - Number of incidents related to the quality management system

## DISCUSSION

The 27 indicators developed in this study provided content evidence and demonstrated that they can be used for nursing management in blood transfusion. Furthermore, based on the adopted framework^(5)^, it was possible to categorize them according to the environment in which they are implemented: structure, process, and outcomes.

For the structure environment, eight indicators were validated, evaluating various aspects, including monitoring nonconformities related to materials and reagents, as well as the preventive and corrective maintenance of critical equipment, proper disposal of blood components, and the number of blood transfusion requests received. Additionally, the training of professionals, the registration of blood components, and the holding of transfusion committee meetings were analyzed.

These indicators allow for the evaluation of physical, human, and organizational conditions, which directly influence the preservation of blood component quality and transfusion safety^(15)^. In this way, an appropriate structure contributes to improving the quality of care, ensuring greater efficacy and safety for the patient.

This statement aligns with the evidence found in the literature regarding the importance of monitoring indicators. For example, the rate of training for professionals involved in blood transfusion is considered crucial for adopting best practices and acquiring skills, as it is well known that professionals’ knowledge in this area is often insufficient, and its absence directly impacts transfusion safety^(16-19)^. Additionally, current legislation requires that professionals receive periodic training on best practices in blood transfusion^(10)^.

Monitoring the disposal of blood components is also an important indicator to be evaluated, as studies have found that the main causes of waste for red blood cell concentrates, platelets, and fresh frozen plasma were expiration dates and units reserved and/or returned from the operating room and/or wards^(20,21)^. In light of this, it is essential to monitor the main causes of blood component disposal, aiming to reduce waste of financial resources and improper treatment of waste^(22)^. According to an estimate by the World Health Organization (WHO), the disposal of red blood cell concentrates represented a loss of approximately 45 million U.S. dollars in Latin American and Caribbean countries in 2011^(23)^.

Regarding the process environment, the eleven validated indicators assess aspects such as the correct completion of blood transfusion requests and the appropriate indication for transfusion according to clinical protocols. In addition, inconsistencies in pre-transfusion tests, blood sample collection, and the release of blood components for transfusion are evaluated, as well as double-checking and monitoring vital signs before infusion. Another aspect assessed was the active search for transfusion reactions. In general, these validated indicators help ensure that blood transfusions are performed correctly and in accordance with safety standards^(15)^.

Through the evaluation of the process, the results of care and the assessment of quality are obtained^(5)^. In blood transfusion, this can be observed when indicators such as incidents related to blood sample collection, pre-transfusion tests, and the release of blood components for transfusion are monitored. Incidents in these activities can lead to severe adverse events, such as acute hemolytic reactions, as well as patient death^(24)^. Therefore, it is necessary to evaluate these indicators so that improvement actions and best practices can be adopted to prevent these occurrences and/or recurrences^(25)^.

When it comes to monitoring the performance of double-checking and verifying vital signs before the installation of blood components, it is known that these actions are required by current legislation^(10)^. However, a study aimed at investigating the execution of the blood transfusion process in relation to compliance with the best practices protocol found that 19% of patients undergoing blood transfusions did not have a record of vital signs before the transfusion started^(26)^. This highlights that, even though mandatory, this activity may not be complied with in some institutions, posing risks to the patient. Regarding double-checking, this strategy is aimed at minimizing errors related to patient identification, thereby maximizing patient safety, and, for this reason, it also needs to be monitored^(27)^.

The assessment of blood transfusion request rates, considering inadequate completion and the alignment of indications with clinical protocols, is an important process indicator that affects safety and the rational use of blood. A study conducted in 2020 analyzed 1,132 blood component requests and found that 4.6% lacked data on the patient’s hemoglobin level, 4.3% were missing information on platelets, 7.1% did not report coagulation time, and 39.6% cited anemia as the sole justification for the transfusion, without additional details^(28)^.

Another study, conducted at a public hospital, performed a similar analysis and observed a significant number of transfusions being performed without a clinical indication, highlighting the irrational use of blood^(29)^. The evidence found in these studies supports the concept of Patient Blood Management (PBM), a globally utilized program that adopts strategies to limit the use and need for blood transfusions, with the goal of improving patient clinical outcomes^(30)^.

In the outcome’s environment, eight indicators were validated, which are directly related to adherence to compatibility recommendations in blood component selection, incidents involving the patient (including preparation, identification, and monitoring), the occurrence of transfusion reactions, incidents in quality management, and the records of blood transfusions both in the patient’s medical chart and in the Transfusion Agency. These indicators not only assess the impact of transfusions but also provide valuable data for improving processes and organizational structure, enhancing transfusion safety, and improving the quality of care^(15)^.

During the selection of blood components for transfusion, compatibility recommendations must be followed to prevent transfusion reactions, such as erythrocyte alloimmunization. In Brazil, 1,058 cases were reported in 2023^(31)^, highlighting that even though legal guidelines exist, compliance with compatibility recommendations is not always observed by institutions, indicating the need for monitoring their implementation.

Regarding indicators that assess incidents related to the patient, these include those associated with preparation, identification, and monitoring, all of which are widely recognized in the literature for their importance. Reports of acute transfusion reactions emphasize the need for close monitoring of patients during transfusion^(24)^. This is particularly critical because, in many cases, patients may exhibit symptoms due to their underlying condition, which can complicate the identification of a reaction.

In addition to monitoring, proper patient identification is one of the activities that must be evaluated through indicators. Errors or near-errors related to patient identification can lead to severe outcomes and require healthcare services to implement measures that influence process reviews, team training, and actions focused on transfusion safety^(32,33)^.

Regarding transfusion reactions, monitoring them helps prevent and mitigate the occurrence or recurrence of unexpected, undesirable, and some even avoidable effects resulting from the therapeutic use of blood components. In the United Kingdom, the incidence of adverse reactions in 2022 was approximately 15 reactions per 10,000 transfused blood components, of which 83.1% were related to avoidable errors; 7.5% were possibly avoidable; and 9.4% were unavoidable. Additionally, 35 deaths related to blood transfusions were reported in 2022^(24)^.

In Brazil, 11,531 confirmed transfusion reactions were reported in 2023. Of these, 79.48% were of mild severity, 16.30% were of moderate severity, 3.73% were severe, and 0.49% were fatalities^(31)^. Failures occurring during blood transfusion can be prevented through investments in training for the professionals involved, revisions of work processes, improvements in management systems, among other measures.

Records related to blood transfusion in the patient’s medical chart and in the Transfusion Agency are critical recommendations outlined in legislation to ensure the traceability of blood components and the transfusion process, verifying that the procedure occurred properly. However, despite being a legal requirement, both national and international studies highlight that failures in transfusion records do exist, which impact the evaluation of the blood transfusion process and the reporting of transfusion reactions^(34-37)^.

Regarding quality management, it is essential in a hemotherapy service, with its implementation being mandated by law in such services^(10)^. Therefore, with the aim of promoting transfusion safety, the National Health Surveillance Agency (ANVISA in Portuguese) conducted an evaluation of hemotherapy institutions in 2022, resulting in the publication of the Annual Health Assessment Bulletin for Hemotherapy Services. The evaluation covered 35.2% of the 2,175 services registered in the national hemotherapy database, identifying 21.39% non-compliance in quality management, notably due to the failure to validate critical processes and the lack of internal audits and standard operating procedures (SOPs) for both technical and administrative aspects^(38)^. This highlights the need to monitor incidents related to quality management to ensure that continuous improvement actions and process control are executed.

In addition to validating the content of the indicators and their technical sheets, the judges assessed the degree of importance of the proposed indicators. Of the 27 indicators, only two were considered unimportant for nursing management in blood transfusion: “Percentage of compatible red blood cell concentrates effectively transfused” and “Number of incidents related to quality management systems”. However, these situations still need to be measured because they have financial impacts on healthcare institutions and the transfusion process, particularly concerning the quality of care provided by professionals, and there are also legal aspects involved^(39-41)^.

Low rates of compatible red blood cell concentrates effectively transfused result in wasted financial and human resources, impacting inventory management and the availability of blood for those who truly need it^(40,41)^. As for incidents related to quality management, it has already been established that this is a legal issue directly connected to blood transfusion safety^(10)^.

Monitoring indicators related to the blood transfusion process is part of the scope of nursing management activities and contributes to the management of best practices and continuous improvement of care. Authors^(42)^ have highlighted that the values measured by these indicators allow for the assessment of the results of the provided care and verification of whether the established goals have been achieved. Furthermore, they contribute to a deeper understanding of the patients served, facilitating the identification of areas for improvement in care. These indicators also support evidence-based planning and decision-making by nurses and help prevent the waste of financial and material resources. By providing a comprehensive view of care, the indicators promote the reduction of risks and harm to patients^(42,43)^.

### Study limitations

A limitation of this study is the lack of published literature on the topic, as this is the first study conducted within the national context, which hindered comparative studies. Additionally, the low level of participation from nurses in the composition of the expert committee during the content validation phase of the indicators is noteworthy.

### Contributions of the study

This study highlights the importance of using indicators for nursing management in blood transfusion. Through this tool, it is possible to assess the care provided by the professionals involved in the transfusion process, identify weaknesses and areas for improvement, adopt best practices to ensure patient safety, reduce material and financial resource wastage, and develop action plans for continuous improvement based on data that reflect the actual situation of the evaluated scenario.

## CONCLUSIONS

The results of this study indicate that the 27 proposed indicators have a valid evidence base for content, making them suitable for use in nursing management throughout the entire transfusion process.

In the context of blood transfusion, the application of these indicators will be essential for evaluating the structural environment, enabling the monitoring of physical, human, and organizational conditions involved in the therapeutic process. They will also facilitate the analysis of the process environment, tracking the care actions provided to patients during the transfusion, including exams and procedures performed. Additionally, these indicators will assist in evaluating the outcome environment, monitoring the effects of care on the patient and ensuring the overall quality of the process.

It is recommended that further studies on indicators for evaluating the transfusion process be conducted in Brazil to foster more in-depth discussions on improving quality in healthcare institutions, adopting best practices in blood transfusion, which will lead to greater safety for blood recipients and improved nursing care.
