# Supplementary material for: Construction and validation of indicators for nursing management in blood transfusion
Source: Rev Bras Enferm. 2025 Jul 11;78(3):e20240229. doi: 10.1590/0034-7167-2024-0229 (PMC12252922; doi:10.1590/0034-7167-2024-0229)
Supplement: Translation

# Translation: Construção e validação de indicadores para a gestão de enfermagem na transfusão de sangue

**DOI:** 10.1590/0034-7167-2024-0229pt

**Authors:** Daiana de Mattia, Dulcinéia Ghizoni Schneider, Francine Lima Gelbcke, Maria Manuela Frederico Ferreira, Edson Pacheco Paladini, Jose Luís Guedes dos Santos, Marcela Ganzella Sisdelli

**Affiliations:** I Universidade Federal de Santa Catarina. Florianópolis, Santa Catarina, Brasil; II Escola Superior de Enfermagem de Coimbra. Coimbra, Portugal; III Fundação Hemocentro de Ribeirão Preto. Ribeirão Preto, São Paulo, Brasil

**Keywords:** Transfusão de Sangue, Cuidados de Enfermagem, Indicadores de Gestão, Estudo de Validação, Serviço de Hemoterapia

## Abstract

**Objetivos:**

construir e validar indicadores para a gestão de enfermagem na transfusão de sangue.

**Métodos:**

estudo metodológico que envolveu a criação das fichas técnicas dos indicadores para a gestão de enfermagem na transfusão de sangue e sua validação por 17 enfermeiros atuantes em agências transfusionais brasileiras. A análise dos dados foi realizada por meio de estatística descritiva, índice de validade de conteúdo e cálculo do percentual de concordância.

**Resultados:**

27 indicadores foram validados para a gestão de enfermagem na transfusão de sangue, com índice de validade de conteúdo superior a 0,80 e percentual de concordância superior a 80%. Esses indicadores foram organizados nas categorias estrutura, processo e resultados, de acordo com o ambiente avaliado.

**Conclusões:**

os indicadores estabelecidos apresentam evidências de validade de conteúdo, permitindo sua aplicação na gestão de enfermagem no processo transfusional, promovendo a melhoria contínua das práticas da equipe de enfermagem e fortalecendo a segurança transfusional.

## INTRODUÇÃO

A qualidade é um conceito dinâmico, de domínio público, que envolve múltiplos elementos com diferentes níveis de importância, cujo objetivo principal é o atendimento das necessidades do consumidor^(1)^. No contexto da saúde, está relacionada à satisfação dos usuários, levando em consideração a prestação de cuidados eficazes e seguros, com um nível de excelência profissional, respeitando os valores culturais e sociais prevalentes^(2)^.

Para atender a esses requisitos, é fundamental realizar avaliações contínuas dos processos de saúde, com o objetivo de identificar os fatores que impactam diretamente o trabalho dos profissionais responsáveis pela assistência^(3)^. Tão importante quanto produzir qualidade é avaliá-la, pois, por meio de criteriosa análise, é possível monitorar as ações em desenvolvimento quanto ao alcance dos objetivos e resultados esperados^(1)^.

A avaliação da qualidade é uma das principais ferramentas para suprir as necessidades de informação dos gestores, sendo essencial para revisar e redirecionar os caminhos seguidos na implementação das ações de saúde^(4)^. Um dos pioneiros na publicação sobre qualidade na saúde criou um modelo conceitual para avaliação, que passou a ser amplamente utilizado nos serviços de saúde, baseado em três ambientes: estrutura, processo e resultados^(5)^.

A estrutura refere-se aos aspectos mais estáveis da produção em saúde, incluindo os recursos físicos, humanos, materiais e financeiros necessários para a prestação de cuidados. O processo envolve as ações realizadas no cuidado, envolvendo tanto os profissionais de saúde quanto os pacientes, sempre com base em padrões definidos. Já o resultado diz respeito aos efeitos da assistência, levando em conta a saúde dos pacientes, o cumprimento de padrões e a satisfação das expectativas^(5)^.

Como ferramenta para avaliação, têm-se os indicadores, que são representações mensuráveis, ou seja, dados quantitativos que refletem as características de produtos e processos, sendo essenciais para monitorar e aprimorar o desempenho e a qualidade das organizações^(1)^. Eles são alicerces para uma análise crítica dos resultados, além de auxiliar tomada de decisões, planejamento e controle dos processos, e direcionam melhorias a serem realizadas^(6)^.

Os processos de gestão da assistência em saúde são amplamente aplicados na prática da enfermagem. No entanto, para que os enfermeiros desenvolvam ferramentas eficazes para avaliar os resultados da assistência, é necessário que sejam fundamentados em dados que traduzam, de maneira direta ou indireta, a realidade desse cuidado^(3)^. Por isso, é essencial que estes profissionais tenham indicadores sensíveis ao seu trabalho, que reflitam a estrutura, processo e resultados dos cuidados prestados^(7)^. Além disso, é fundamental fortalecer a cultura da qualidade nos serviços, promovendo a capacitação para a criação e análise de indicadores o que possibilita uma avaliação contínua e dinâmica da assistência, com foco na segurança do paciente, redução de eventos adversos e aprimoramento dos processos de gestão^(8)^.

Autores^(9)^ afirmam que o enfermeiro na hemoterapia necessita desempenhar um importante papel no atendimento do receptor de sangue, seja na busca constante em disponibilizar serviços e produtos de qualidade, assim como no atendimento assistencial. Esta atuação também permeia a produção dos hemocomponentes, no desenvolvimento do ensino e da pesquisa, reduzindo a distância entre a prática e o conhecimento científico.

Legalmente os serviços de hemoterapia no Brasil devem implementar indicadores e metas para monitorar o desempenho dos processos ao longo de todo o ciclo do sangue, desde a coleta até a transfusão^(10)^. No entanto, um estudo realizado demonstrou que há uma escassez de indicadores voltados à assistência prestada aos pacientes submetidos à transfusão de sangue, tanto no contexto nacional quanto internacional, o que evidencia a necessidade de aprofundar os estudos sobre essa questão^(11)^.

## OBJETIVOS

Construir e validar indicadores para a gestão de enfermagem na transfusão de sangue.

## MÉTODOS

### Aspectos éticos

O estudo foi conduzido de acordo com as diretrizes éticas estabelecidas pelos órgãos reguladores nacionais e internacionais e foi aprovado pelo Comitê de Ética em Pesquisa da Universidade Federal de Santa Catarina e do Centro de Pesquisa Oncológica (CEPON). Todos os participantes assinaram o Termo de Consentimento Livre e Esclarecido (TCLE).

### Desenho, período e local de estudo

Trata-se de um estudo metodológico de construção e validação de indicadores para a gestão de enfermagem no processo transfusional, desenvolvido de acordo com os procedimentos metodológicos propostos por McGlynn e Asch^(12)^, seguindo quatro etapas: escolha das áreas de avaliação; seleção dos indicadores; projeção das especificações para uma medida; e, testagem da força científica da medida.

A coleta dos dados ocorreu entre fevereiro a abril de 2023, de forma *on-line*, em Agências Transfusionais localizadas nas cinco regiões do Brasil.

### População, critérios de inclusão e exclusão

Foram convidados, intencionalmente, a compor o painel de juízes especialistas, 174 enfermeiros que atuavam em Agências Transfusionais de cinco regiões do Brasil. Como critérios de inclusão no estudo, consideraram-se enfermeiros que estavam em atividade há pelo menos um ano na Agência Transfusional. Em relação aos critérios de exclusão, foram preteridos os enfermeiros que estavam afastados por atestados ou em férias no período da coleta dos dados.

### Protocolo do estudo

As duas primeiras etapas do estudo, escolha das áreas de avaliação e a seleção dos indicadores de desempenho, foram descritas em dois manuscritos, sendo que um já está publicado em periódico nacional^(11)^. A partir dessas etapas, foram definidos 27 indicadores para a gestão de enfermagem na transfusão de sangue.

Com indicadores definidos, seguiu-se para a terceira etapa onde foram projetadas as especificações para uma medida a partir da construção das fichas técnicas dos indicadores, adaptada do modelo recomendado por Paladini^(1)^, que contém os seguintes itens: objetivo - determina o que deverá ser avaliado pelo indicador; justificativa - refere-se à importância de se proceder a avaliação proposta pelo indicador; ambiente -trata da natureza do indicador; padrão - que é o referencial utilizado para avaliar se o processo em questão apresentou melhorias; descrição - que se refere ao contexto, às situações, ou à natureza que basicamente caracteriza o indicador; fator - a forma como se dá o cálculo do indicador; medida - a unidade com a qual se medem os fatores, envolvendo o sistema internacional de medidas e a temporalidade de verificação.

Na quarta etapa, determinou-se a testagem da força científica da medida por meio da validade de conteúdo. Para a coleta dos dados foi construído um formulário *on-line*, na plataforma *Google Forms^®^,* e seu *link* encaminhado por *e-mail*, com uma carta convite. O formulário foi dividido nas seguintes sessões: 1) TCLE, para leitura e confirmação do aceite de participação na pesquisa; 2) Caracterização dos juízes especialistas, sendo composta de 14 questões sobre o profissional e seu local de trabalho; 3) Avaliação dos 27 indicadores e suas respectivas fichas técnicas.

Na sessão 3 foram utilizadas questões fechadas para que os juízes avaliassem a ortografia, pertinência, objetividade, clareza, precisão, viabilidade, representatividade, visualização, ajuste, unicidade, alcance e resultados, conforme recomenda Paladini^(1)^. Além disso, foi avaliada, também, a importância deste indicador para a gestão de enfermagem.

O questionário foi elaborado com escala de respostas tipo *Likert* de quatro pontos (Discordo totalmente - Concordo totalmente). Ainda, foi reservado um espaço para que cada participante pudesse deixar comentários e/ou sugestões para refinamento dos indicadores.

Na primeira rodada, 17 juízes enviaram o questionário preenchido no prazo estabelecido de trinta dias. Para a segunda rodada, reformulou-se o conteúdo de nove indicadores e um novo indicador foi construído, conforme recomendações dos juízes. O formulário, contemplando as modificações sugeridas, foi encaminhado ao mesmo grupo de juízes, com prazo de vinte dias para devolução. Fizeram parte dessa etapa sete juízes, os quais puderam, novamente, apresentar sugestões e observações pertinentes para a melhoria dos indicadores.

### Análise dos resultados e estatística

O conjunto de dados enviados pelos juízes foi armazenado em uma planilha do programa *Microsoft Excel* ^®^ para análises posteriores, as quais foram executadas através do programa *Statistical Package for the Social Sciences* (SPSS) versão 23 (IBM Corporation, Armonk, NY, USA).

A normalidade dos dados foi verificada pelo teste de Shapiro-Wilk. Para as variáveis numéricas, foi utilizada estatística descritiva, incluindo medidas de tendência central e dispersão, enquanto as variáveis categóricas foram apresentadas por meio de frequências absolutas e relativas.

Para avaliar o consenso entre os juízes especialistas nas duas rodadas, foram utilizados dois métodos: o Índice de Validade de Conteúdo (IVC) e o Percentual de Concordância. O primeiro foi calculado por meio da razão da soma das respostas concordantes, ou seja, respostas “3” e “4” pelo número total de juízes participantes. Já o segundo, foi calculado através da razão entre a soma das respostas concordantes pelo número total de juízes participantes multiplicados por 100. A literatura recomenda que, tanto o item quanto o questionário total, para serem considerados aceitáveis, se faz necessário alcançar IVC de 0,8 e 80% de concordância entre os participantes^(13,14)^.

## RESULTADOS

Os dados da primeira rodada foram obtidos a partir de um painel de juízes especialistas composto por 17 enfermeiros. A idade mínima registrada foi de 28 anos e a máxima de 55 anos, com média de 41,0 (DP = 7,9) anos. Quatorze (82,4%) tinham formação em Enfermagem; dois (11,8%), em Enfermagem e Obstetrícia; e um (5,9%), em Enfermagem e Nefrologia. Onze possuíam título de especialização e seis (35,3%) tinham título de mestrado. A média de tempo de formação acadêmica foi de 14,6 (DP: 10,0) anos, e o tempo de atuação em agência transfusional apresentou mediana de 4,0 (2,0 - 8,0) anos.

Referente às características das Agências Transfusionais em que os juízes trabalham, destacam-se alguns dados, como: oito (47,1%) eram da região Sul; 14 (82,4%) de modalidade pública; dez (58,8%) tinham gestão realizada pelo Hospital/Clínica; e, 11 (64,7%) Agências realizavam de 100 a 500 transfusões de sangue por mês. Sete (41,2%) possuíam alguma acreditação, entre as quais: a *Joint Comition International, International Organization for Standardization (*ISO) 9001; Acreditação Nacional; duas agências possuíam mais de uma acreditação; duas tinham a ISO e *American Association of Blood Banks* (AABB); e, 15 (88,2%) utilizavam algum tipo de indicador para avaliar o processo de trabalho.

Todos os indicadores apresentaram um IVC total acima de 0,80, sendo a variação de 0,85 a 1,00 para os itens avaliados. Em relação ao percentual de concordância, todos os indicadores atingiram mais de 80% de concordância entre os juízes especialistas, variando entre 85% e 100%. No que diz respeito ao grau de importância, dos 27 indicadores, 17 foram avaliados como muito importantes ou importantes, 10 foram considerados pouco importantes por um ou dois juízes e apenas dois foram classificados sem importância por um juiz.

Os juízes emitiram alguns comentários e sugestões, que foram analisados, sendo concluído que nove indicadores precisavam ser refinados.

Tendo em vista essa reformulação, apesar dos indicadores terem alcançado um IVC >0,80 e PC > 80%, optou-se pela realização de uma segunda rodada com o painel de juízes especialistas para avaliar o índice de validade de conteúdo dos indicadores revisados e do novo indicador construído. Sete enfermeiros participaram da segunda rodada, os quais avaliaram novamente: Concordância e Ortografia; Pertinência; Objetividade; Clareza; Precisão; Viabilidade; Representatividade; Visualização; Ajuste; Unicidade; Alcance; e, Resultados dos dez indicadores reformulados.

O IVC total dos indicadores foi maior que 0,80, variando entre 0,93 a 1,00 e o percentual de concordância de 93% a 100%, demonstrando que todos os indicadores alcançaram percentual acima do mínimo aceitável de acordo com o que preconiza a literatura^(13,14)^.

Alguns participantes teceram comentários e sugestões acerca de determinados indicadores, contudo, estas não foram acatadas, pois iam de encontro à legislação vigente, descaracterizavam o objetivo do indicador proposto ou havia um indicador para a sugestão já apresentada.

Quanto a um novo indicador, o mesmo foi avaliado em relação ao seu grau de importância para a gestão de enfermagem no processo transfusional e foi considerado relevante pelos sete juízes que o avaliaram.

Ao final da segunda rodada, foram validados 27 indicadores para a gestão de enfermagem, os quais estão organizados de acordo com o ambiente^(5)^ e descritos no Quadro 1.

**Quadro 1:** Indicadores validados para a gestão de enfermagem na transfusão de sangue, Florianópolis, Santa Catarina, Brasil, 2024

AMBIENTE	INDICADORES
**Estrutura**	Indicador 1 - Número de solicitações de transfusão de hemocomponentes recebidas pela agência transfusional
	Indicador 8 - Taxa de descarte de hemocomponentes durante inspeção visual
	Indicador 21 - Taxa de descarte de hemocomponentes na agência transfusional
	Indicador 22 - Número de não conformidades identificadas na avaliação da qualidade dos materiais e reagentes
	Indicador 23 - Taxa de equipamentos críticos com qualificação, calibração e manutenção preventiva realizada
	Indicador 24 - Taxa de profissionais que receberam treinamento sobre o processo de transfusão de sangue na instituição
	Indicador 25 - Taxa de hemocomponentes sem registro de destino final
	Indicador 26 - Número de reuniões realizadas pelo comitê transfusional
**Processo**	Indicador 2 - Taxa de solicitação de transfusão de hemocomponentes recebidas pela agência transfusional com preenchimento inadequado.
	Indicador 3 - Taxa de solicitação de transfusão de hemocomponentes recebidas que não estão de acordo com a indicação clínica de cada hemocomponente
	Indicador 4 - Número de discrepâncias entre os resultados de tipagem sanguínea encontradas nos exames pré-transfusionais e os resultados registrados no histórico do paciente
	Indicador 5 - Taxa de incidentes relacionados às amostras de sangue para os exames pré-transfusionais
	Indicador 6 - Taxa de incidentes relacionados aos exames pré-transfusionais
	Indicador 9 - Taxa de resultado tipagem sanguínea discrepante encontrado na retipagem dos concentrados de hemácias liberados para transfusão
	Indicador 10 - Índice de concentrado de hemácia compatibilizados e efetivamente transfundidos
	Indicador 11 - Número de hemocomponentes com inconsistências na etiqueta de liberação
	Indicador 13 - Taxa de transfusão de sangue sem registro de dupla checagem antes da instalação do hemocomponente
	Indicador 14 - Taxa de transfusões realizadas sem verificação dos sinais vitais antes do início da transfusão
	Indicador 27 - Taxa de busca ativa de reações transfusionais
**Resultado**	Indicador 7 - Taxa de cumprimento das recomendações de compatibilidade durante a seleção de hemocomponentes
	Indicador 12 - Taxa de incidentes relacionados ao preparo do paciente para transfusão de sangue
	Indicador 15 - Taxa de incidentes relacionados à identificação inadequada do paciente
	Indicador 16 - Taxa de pacientes que não foram monitorados na transfusão de sangue
	Indicador 17 - Índice de reação transfusional por tipo de reação, gravidade, por tipo de hemocomponente e tempo de aparecimento
	Indicador 18 - Taxa de não conformidades relacionadas aos registros da transfusão de sangue no prontuário do paciente
	Indicador 19 - Taxa de não conformidades relacionadas aos registros da transfusão de sangue na agência transfusional
	Indicador 20 - Número de incidentes relacionados ao sistema da gestão da qualidade

## DISCUSSÃO

Os 27 indicadores construídos neste estudo apresentaram fontes de evidência de conteúdo e demonstraram que podem ser utilizados para a gestão de enfermagem na transfusão de sangue. Ainda, com base no referencial adotado^(5)^, foi possível categorizá-los de acordo com o ambiente em que estão inseridos: estrutura, processo e resultados.

Para o ambiente estrutura, foram validados oito indicadores que avaliam diversos aspectos, incluindo o monitoramento das não conformidades relacionadas aos materiais e reagentes, bem como da manutenção preventiva e corretiva dos equipamentos críticos, o descarte adequado de hemocomponentes e o número de solicitações de transfusão de sangue recebidas. Além disso, são analisados o treinamento dos profissionais, o registro dos hemocomponentes e a realização de reuniões do comitê transfusional.

Esses indicadores possibilitam a avaliação das condições físicas, humanas e organizacionais, que influenciam diretamente na preservação da qualidade dos hemocomponentes e na segurança da transfusão de sangue^(15)^. Dessa forma, uma estrutura apropriada contribui para a melhoria da qualidade da assistência, garantindo maior eficácia e segurança para o paciente.

Tal afirmativa vai ao encontro das evidências encontradas na literatura que tratam da importância de monitorar indicadores. Como exemplo, tem-se a taxa de treinamento dos profissionais envolvidos na transfusão de sangue, sendo este apontado como primordial para a adoção de melhores práticas e aquisição de habilidades, pois se sabe que o conhecimento dos profissionais é deficiente nesta área e a falta dele implica diretamente na segurança transfusional^(16-19)^. Somado a este fato, a legislação vigente exige que os profissionais recebam treinamento periódico sobre as boas práticas na transfusão de sangue^(10)^.

O monitoramento do descarte de hemocomponentes também é um importante indicador a ser avaliado, pois estudos constataram que as principais causas de desperdício de concentrado de hemácias, plaquetas e plasma fresco congelado foram o prazo de validade e unidades reservadas e/ou devolvidas da sala de cirurgia e/ou enfermaria^(20,21)^. Diante dessa questão, é essencial monitorar as principais causas do descarte de hemocomponentes, visando reduzir o desperdício de recursos financeiros e o tratamento inadequado de resíduos^(22)^. De acordo com uma estimativa da Organização Mundial da Saúde (OMS), o descarte de concentrado de hemácias representou uma perda de aproximadamente 45 milhões de dólares americanos nos países da América Latina e Caribe, em 2011^(23)^.

No que se refere ao ambiente processo, os onze indicadores validados avaliam aspectos como o preenchimento correto das solicitações de transfusão de hemocomponentes e a adequada indicação da transfusão, conforme os protocolos clínicos. Também são analisadas as inconsistências nos exames pré-transfusionais, a coleta de amostras de sangue e a liberação dos hemocomponentes para transfusão de sangue, além da dupla checagem e monitoramento dos sinais vitais antes da infusão. Outro ponto avaliado foi a busca ativa das reações transfusionais. De modo geral, estes indicadores validados ajudam a assegurar que a transfusão de sangue seja realizada de forma correta e dentro dos padrões de segurança^(15)^.

Por meio da avaliação do processo, obtêm-se os resultados da assistência e a valoração da qualidade^(5)^. Na transfusão de sangue, esta afirmação pode ser observada quando são monitorados indicadores como incidentes relacionados à coleta de amostra de sangue, aos exames pré-transfusionais e à liberação do hemocomponente para transfusão, por exemplo. Incidentes nestas atividades podem levar a eventos adversos graves, como as reações hemolíticas agudas, bem como a morte do paciente^(24)^. Por isso, é necessário que eles sejam avaliados para que ações de melhoria e boas práticas sejam adotadas, a fim de evitar essas ocorrências e/ou recorrências^(25)^.

Quando se trata de monitorar a realização da dupla checagem e da verificação dos sinais vitais antes da instalação dos hemocomponentes, sabe-se que são condutas exigidas pela legislação vigente^(10)^. No entanto, um estudo cujo objetivo foi investigar a execução do processo de transfusão de sangue em relação ao cumprimento do protocolo de boas práticas identificou que 19% dos pacientes submetidos à transfusão de sangue não apresentaram registro de sinais vitais antes do início da transfusão^(26)^. Isso evidencia que, mesmo obrigatória, essa atividade pode não ser cumprida nas instituições, acarretando riscos para o paciente. No que diz respeito à dupla checagem, esta é uma estratégia que objetiva minimizar erros relacionados à identificação do paciente, maximizando sua segurança e, por isso, também necessita ser monitorada^(27)^.

A avaliação da taxa de solicitações de transfusão de hemocomponentes, considerando o preenchimento inadequado e a conformidade da indicação com os protocolos clínicos, é um importante indicador de processo que implica na segurança e no uso racional do sangue. Uma pesquisa realizada em 2020 analisou 1.132 solicitações de hemocomponentes e constatou que 4,6% não apresentavam dados sobre o nível de hemoglobina do paciente, 4,3% estavam sem informações sobre as plaquetas, 7,1% não informavam o tempo de coagulação, e 39,6% indicavam como justificativa para a transfusão apenas anemia, sem detalhes adicionais^(28)^.

Outro estudo, realizado em um hospital público, fez uma análise semelhante e observou uma quantidade significativa de casos de transfusão sem indicação clínica, constatando o uso irracional do sangue^(29)^. As evidências encontradas nesses estudos vão ao encontro da proposta do *Patient Blood Management* (PBM), programa mundialmente utilizado e que adota estratégias para limitar o uso e a necessidade de transfusão de sangue, com o objetivo de melhorar os resultados clínicos do paciente^(30)^.

Para o ambiente de resultados, foram validados oito indicadores, os quais estão diretamente relacionados ao cumprimento das recomendações de compatibilidade na seleção de hemocomponentes, aos incidentes envolvendo o paciente (incluindo preparo, identificação e monitoramento), à ocorrência de reações transfusionais, aos incidentes na gestão da qualidade e aos registros da transfusão de sangue tanto no prontuário do paciente quanto na Agência Transfusional. Esses indicadores não apenas avaliam o impacto das transfusões, mas também fornecem dados valiosos para aprimorar os processos e a estrutura organizacional, promovendo a segurança transfusional e elevando a qualidade do cuidado prestado^(15)^.

Durante a seleção dos hemocomponentes para a transfusão, as recomendações de compatibilidade devem ser seguidas a fim de evitar a ocorrência de reações transfusionais, como é o caso da aloimunização eritrocitária. No Brasil, foram notificados, em 2023, 1.058 casos^(31)^, evidenciando que, mesmo havendo diretrizes legais, o cumprimento das recomendações de compatibilidade nem sempre é seguido pelas instituições, indicando a necessidade de se monitorar sua execução.

No que diz respeito aos indicadores que avaliam os incidentes relacionados ao paciente, encontram-se os associados ao preparo, à identificação e à monitorização, sendo evidente na literatura sua importância. Relatos de reações transfusionais agudas enfatizam a necessidade de monitorar os pacientes de perto durante a transfusão^(24)^. Isso é especialmente importante porque, em muitos casos, os pacientes podem apresentar sintomas devido à sua condição subjacente, o que pode dificultar a identificação de uma reação.

Além do monitoramento, a identificação adequada do paciente é uma das atividades que precisam ser avaliadas por meio de indicadores. Erros ou quase erros relacionados à identificação do paciente podem resultar em desfechos graves e exigem dos serviços medidas que impactam a revisão dos processos, o treinamento de toda a equipe e ações voltadas para a segurança na transfusão de sangue^(32,33)^.

Frente às reações transfusionais, monitorá-las permite prevenir e mitigar a ocorrência ou a recorrência de efeitos inesperados, indesejáveis e alguns até mesmo evitáveis, resultantes do uso terapêutico de hemocomponentes. No Reino Unido, a incidência de reações adversas, em 2022, foi de aproximadamente 15 reações por 10.000 hemocomponentes transfundidos, das quais 83,1% foram relacionadas a erros evitáveis; 7,5%, possivelmente evitáveis; 9,4%, não evitáveis. Além disso, foram notificadas 35 mortes relacionadas à transfusão de sangue em 2022^(24)^.

No Brasil, em 2023, foram notificadas 11.531 reações transfusionais confirmadas. Destacam-se as reações de gravidade leve, com 79,48%; seguidas das de gravidade moderada, com 16,30%; gravidade grave, 3,73%; e óbitos, em 0,49% dos casos^(31)^. Falhas cometidas na transfusão de sangue podem ser evitadas por meio de investimentos na formação dos profissionais envolvidos, revisão dos processos de trabalho, melhoria nos sistemas de gestão, entre outros.

Os registros relacionados à transfusão de sangue no prontuário do paciente e na Agência Transfusional são recomendações importantes da legislação para garantir a rastreabilidade do hemocomponente e do processo transfusional, observando se transcorreu de forma adequada. Contudo, mesmo sendo uma exigência legal, há estudos nacionais e internacionais que destacam que falhas nos registros transfusionais existem e que implicam na avaliação do processo de transfusão de sangue e na notificação das reações transfusionais^(34-37)^.

No que tange à gestão da qualidade, esta é primordial em um serviço de hemoterapia, sendo previsto por lei sua implantação nesses serviços^(10)^. Por isso, com o objetivo de promover a segurança transfusional, a Agência Nacional de Vigilância Sanitária (ANVISA) realizou, em 2022, uma avaliação das instituições hemoterápicas, resultando na publicação do Boletim Anual de Avaliação Sanitária em Serviços de Hemoterapia. A avaliação cobriu 35,2% dos 2.175 serviços registrados no cadastro nacional de hemoterapia, identificando 21,39% de não conformidades na gestão da qualidade, com destaque para a não validação de processos críticos e a falta de auditorias internas e procedimentos operacionais padrão (POP) técnicos e administrativos^(38)^. Isso mostra a necessidade de acompanhar os incidentes voltados à gestão da qualidade para que ações de melhoria contínua e controle do processo sejam executadas.

Além da validação do conteúdo dos indicadores e suas fichas técnicas, os juízes avaliaram o grau de importância dos indicadores propostos. Dos 27, apenas dois foram considerados sem importância para a gestão de enfermagem na transfusão de sangue: “Índice de concentrado de hemácias compatibilizados e efetivamente transfundidos” e “Número de incidentes relacionados ao sistema da gestão da qualidade”. Contudo, essas situações necessitam ser mensuradas, pois geram impactos financeiros às instituições de saúde e no processo transfusional, principalmente na qualidade prestada pelos profissionais, bem como há aspectos legais relacionados^(39-41)^.

Quando se têm baixos índices de concentrado de hemácias compatibilizados e efetivamente transfundidos, resulta em desperdício de recursos financeiros e humanos, repercute na gestão de estoque e na disponibilização de sangue para quem realmente necessita^(40,41)^. No que concerne aos incidentes da gestão da qualidade, já foi visto que esta é uma questão legal e que há relação direta com a segurança da transfusão de sangue^(10)^.

Realizar o monitoramento dos indicadores voltados ao processo de transfusão de sangue faz parte do escopo das atividades gerenciais da enfermagem e contribui para a gestão de boas práticas e para a melhoria contínua do cuidado. Autores^(42)^ destacaram que os valores mensurados pelos indicadores permitem avaliar os resultados da assistência oferecida e verificar se as metas estabelecidas foram atingidas. Além disso, contribuem para uma compreensão mais aprofundada dos pacientes atendidos, facilitando a identificação de áreas de melhoria no cuidado. Eles também apoiam o planejamento e a tomada de decisões pelos enfermeiros, baseados em evidências, e ajudam a evitar desperdícios de recursos financeiros e materiais. Ao proporcionar uma visão abrangente do cuidado, os indicadores promovem a redução de riscos e danos aos pacientes^(42,43)^.

### Limitações do estudo

Como limitação do estudo, considera-se o fato de haver pouca literatura publicada sobre o tema, sendo este o primeiro a ser realizado no cenário nacional, o que inviabilizou estudos comparativos. Além disso, destaca-se a baixa adesão dos enfermeiros na composição do comitê de especialistas na etapa de validação de conteúdo dos indicadores.

### Contribuições do estudo

Com este estudo, foi possível constatar o quão importante é o uso de indicadores para a gestão de enfermagem na transfusão de sangue. A partir dessa ferramenta, é possível avaliar a assistência prestada pelos profissionais envolvidos no processo transfusional, identificar as fragilidades e pontos de melhoria, adotar melhores práticas, visando à segurança do paciente, à diminuição de desperdícios de recursos materiais e financeiros, e à construção de planos de ação para a melhoria contínua, com base em dados que expressam a situação real do cenário avaliado.

## CONCLUSÕES

Os resultados do presente estudo apontam que os 27 indicadores propostos possuem fonte de evidência de validade de conteúdo, sendo, portanto, apropriados para a utilização na gestão de enfermagem em todo o processo transfusional.

No contexto da transfusão de sangue, a aplicação desses indicadores será essencial para a avaliação do ambiente de estrutura, permitindo o monitoramento das condições físicas, humanas e organizacionais envolvidas no processo terapêutico. Eles também viabilizarão a análise do ambiente de processo, acompanhando as ações de cuidado prestadas aos pacientes durante a transfusão, incluindo exames e procedimentos realizados. Adicionalmente, esses indicadores auxiliarão na avaliação do ambiente de resultado, monitorando os efeitos da assistência sobre o paciente e assegurando a qualidade do processo como um todo.

Sugere-se que mais estudos sobre indicadores para avaliação do processo transfusional sejam realizados no Brasil, de forma a fomentar com maior propriedade discussões sobre a melhoria da qualidade nas instituições de saúde, a adoção de melhores práticas na transfusão de sangue, repercutindo em maior segurança ao receptor de sangue e na qualidade da assistência de enfermagem.
